# Supplementary material for: Innate Cytokine Induced Early Release of IFNγ and CC Chemokines from Hypoxic Human NK Cells Is Independent of Glucose
Source: Cells. 2020 Mar 17;9(3):734. doi: 10.3390/cells9030734 (PMC7140646; doi:10.3390/cells9030734)
Supplement: Supplementary file 1 [file cells-09-00734-s001.pdf]

SUPPLEMENTARY MATERIAL

		Page
**Supplemental Figure S1**	Glycolytic parameters in human NK cells primed with IL-15 under chemical hypoxia.	2
**Supplemental Figure S2**	Flow cytometric gating strategy for IFNγ quantitation.	3
**Supplemental Figure S3**	Flow cytometric gating strategy for the quantitation of multiple markers.	4
**Supplemental Figure S4**	Glycolytic parameters in untreated and IL-15 primed human NK cells.	5
**Supplemental Figure S5**	Effect of DMOG on glycolytic parameters in IL-15/IL-12/ IL-18 treated human NK cells.	6
**Supplemental Figure S6**	Glucose deprivation has little effect on stable oxygen consumption in human NK cells.	6
**Supplemental Figure S7**	Early cytokine responses in human NK cells to IL-12/IL-18 after long-term IL-15 exposure do not require glucose.	7
**Supplemental Figure S8**	DMOG is detrimental to IFNγ production and CC chemokine release by IL-15 primed human NK cells in response to IL-12/IL-18.	8
**Supplemental Discussion**		9

Supplemental Figure S1

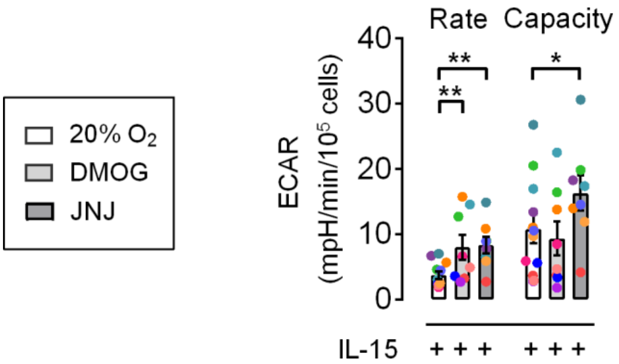

Glycolytic parameters in human NK cells primed with IL-15 under chemical hypoxia. Freshly isolated human NK cells from 8–12 donors were cultured in the presence of 20 µM DMOG or 50 µM JNJ for 16 h. IL-15 was added for another 6 h. Glycolytic rate and capacity were determined using the Seahorse XF Glycolysis Stress Test kit. Data is shown as mean values ± SEM (*bars*) and scatter plots in a color scheme to identify data from same donors, i.e., independent experiments. Statistical significance of mean differences was determined with the Wilcoxon signed-rank test for pairwise comparisons. * $p < 0.05$, ** $p < 0.01$.

Supplemental Figure S2

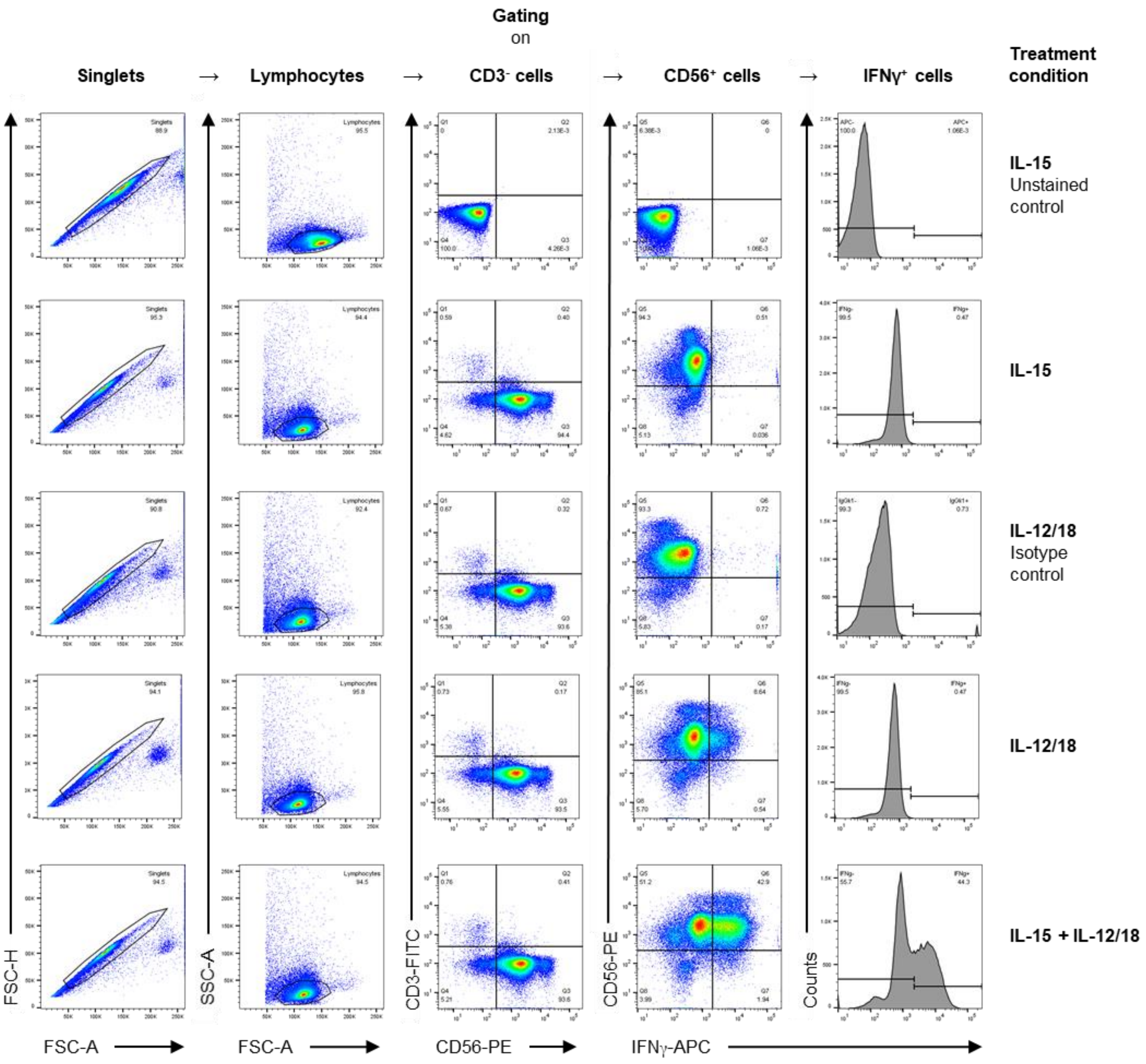

Flow cytometric gating strategy for IFNγ quantitation. Representative dot plots and histograms illustrating the flow cytometric gating strategy used for IFNγ quantitation in NK cells used in Figure 2 of the main text. An example for scheme 1 is shown. Following sequential gating on singlets and lymphocytes, NK cells were defined as $CD3^-$ $CD56^+$ events and represented 100% of the target population for which the percentage and MeFI of IFNγ positive cells were determined (histogram). For cells treated with IL-15 alone and with IL-12 and IL-18 combined (IL-12/18), an unstained control and an isotype-matched control (IgG1κ) is shown, respectively, confirming separation of IFNγ expressing cells from background and non-specific APC fluorescence.

Supplemental Figure S3

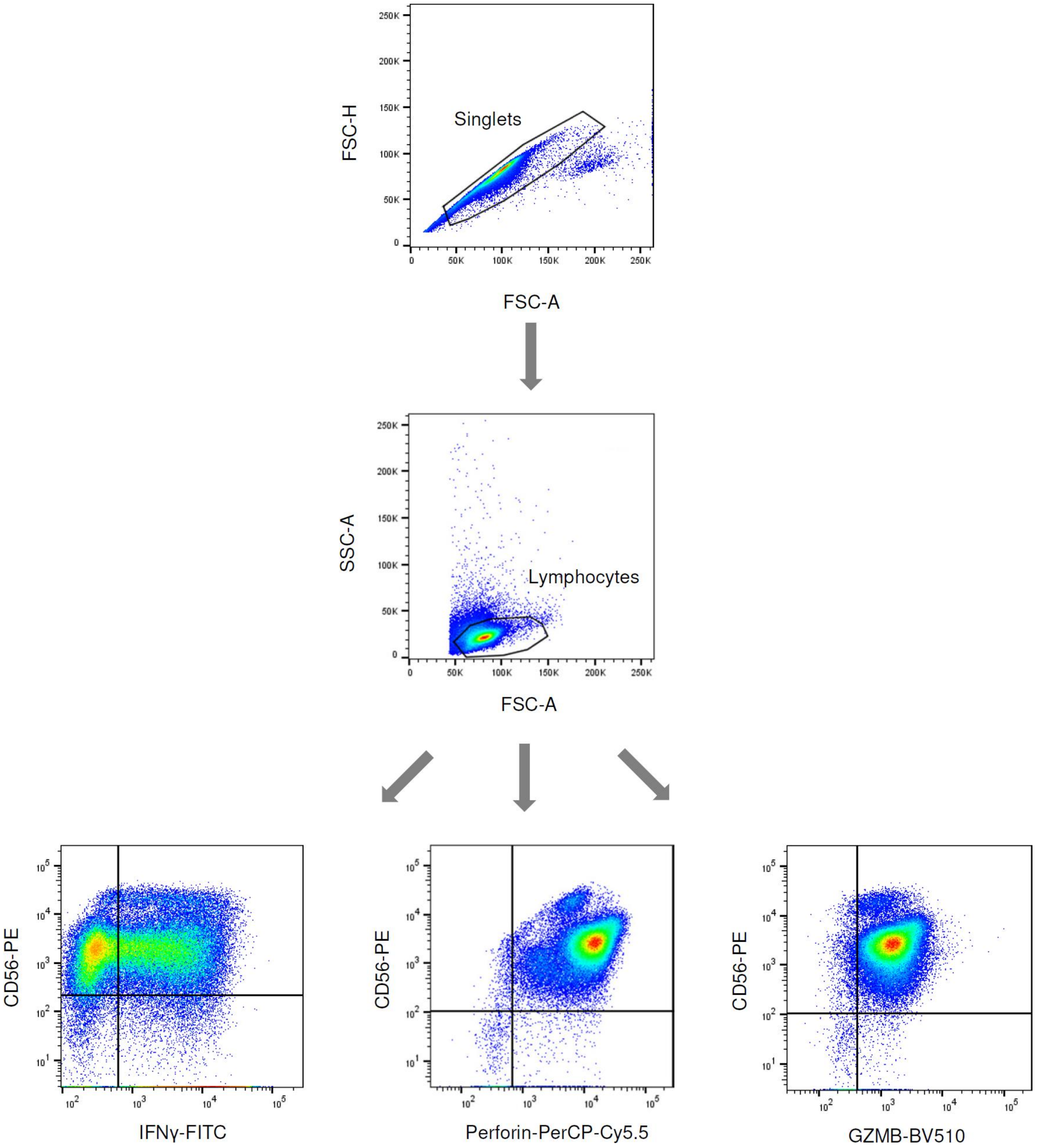

Flow cytometric gating strategy for the quantitation of multiple markers. The strategy for identifying marker positive cell populations in isolated NK cells is exemplified for the effector markers IFNγ, perforin, and granzyme B (GZMB) in cells treated with IL-15 and IL-12/IL-18 according to scheme 1 (Figure 1 in the main text). Following sequential gating on singlets and lymphocytes, events in the upper left quadrant were counted as marker positive NK cells. The population positive for the metabolic marker GLUT1 was quantitated accordingly. To calculate the proportion of marker positive cells, the sum of events in both upper quadrants was set to 100%.

Supplemental Figure S4

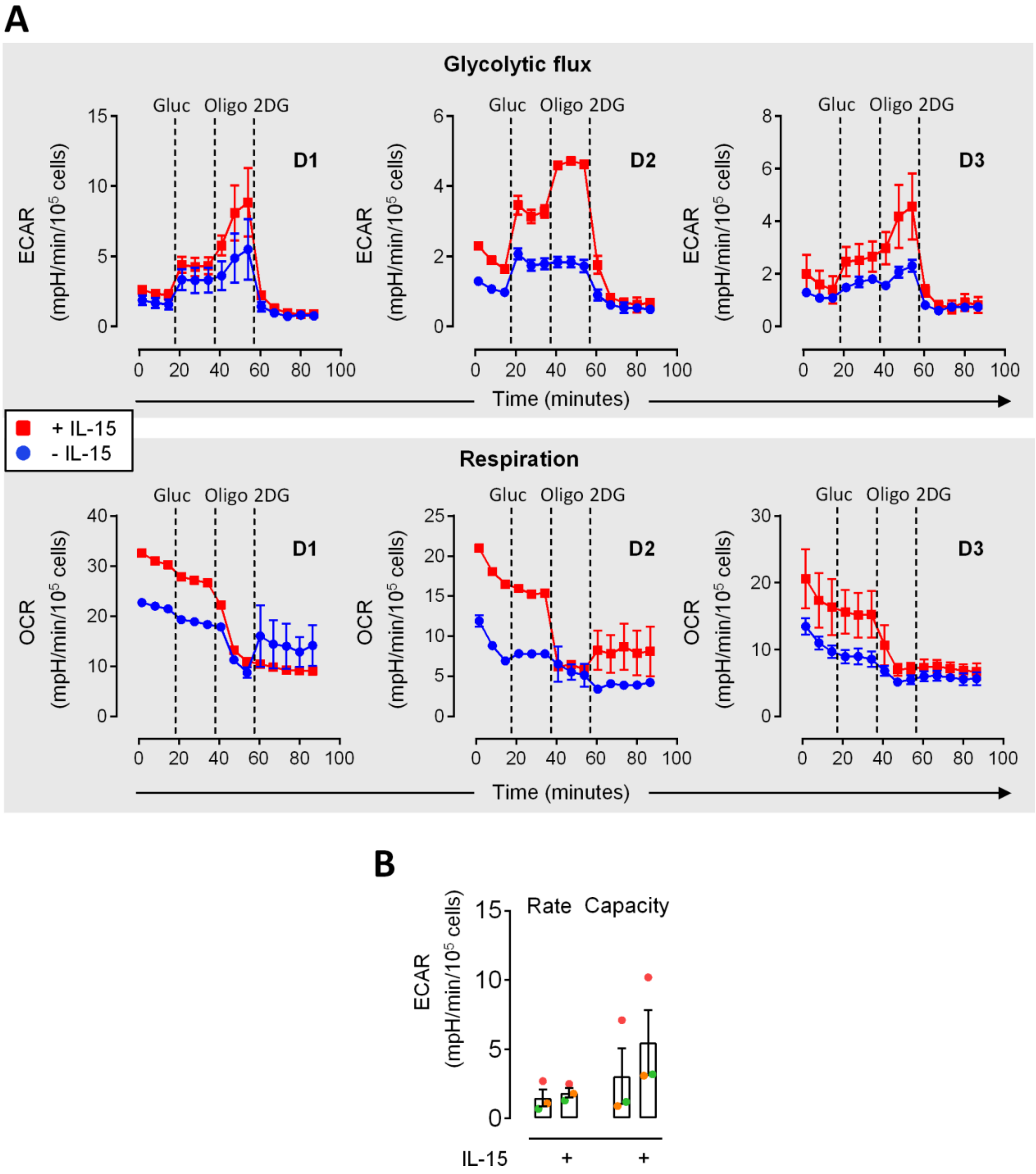

Glycolytic parameters in untreated and IL-15 primed human NK cells. Freshly isolated human NK cells from 3 donors were cultured under standard conditions for 16 h. For priming, IL-15 was added for another 6 h. Glycolytic rate and capacity were determined by adding 10 mM glucose and 1 µM oligomycin, respectively, using the Seahorse XF Glycolysis Stress Test kit. Data is shown (**A**) as mean values ± SEM (bars) and (**B**) scatter plots in a color scheme to identify data from same donors, i.e., independent experiments. Data for both conditions in panel **B** were compared with the Wilcoxon signed-rank test, but the level of statistical significance was not reached.

Supplemental Figure S5

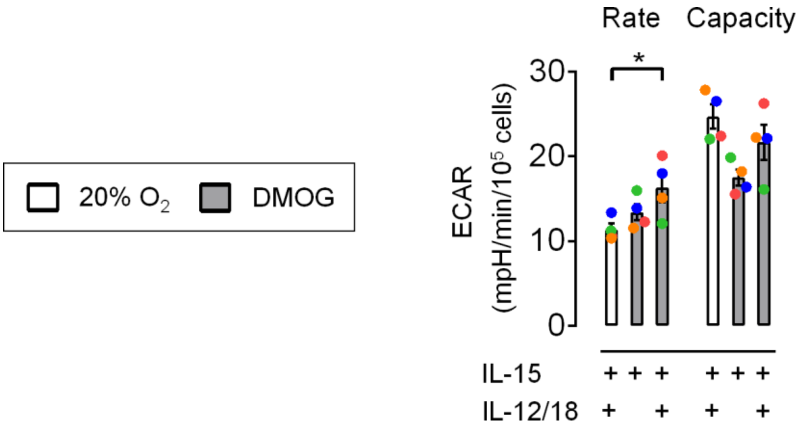

Effect of DMOG on glycolytic parameters in IL-15/IL-12/IL-18 treated human NK cells. Freshly isolated human NK cells from 4 donors were cultured in the presence of DMOG for 16 h. IL-15 was added for another 6 h followed by combined addition of IL-12 and IL-18 (IL-12/18) for further 4 h. Glycolytic rate and capacity were determined using the Seahorse XF Glycolysis Stress Test kit. Data is shown as mean values ± SEM (bars) and scatter plots in a color scheme to identify data from same donors, i.e., independent experiments. Statistical significance of mean differences was determined with the Friedman test with Dunn's test for post-hoc pairwise comparisons. * $p < 0.05$.

Supplemental Figure S6

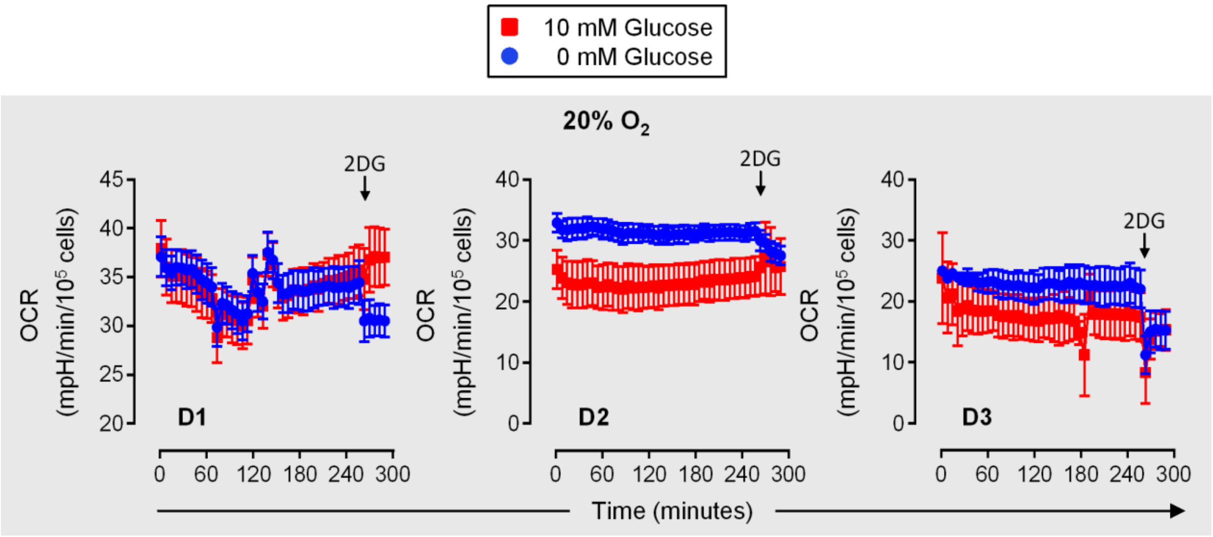

Glucose deprivation has little effect on stable oxygen consumption in human NK cells. The oxygen consumption rate (OCR) traces shown correspond to the simultaneous extracellular acidification rate (ECAR) traces under 20% O_2 in Figure 6 of the main text (D1–D3). NK cells were isolated and cultured under normoxia (20% O_2). At 16 h, IL-15 was added for another 6 h. At 22 h cells were transferred into glucose-containing (red) or glucose-deficient (blue) medium in the continued presence of IL-15 plus IL-12/IL-18 and were immediately subjected to simultaneous ECAR and OCR measurements. Toward the end of the experiment, glycolysis was inhibited by adding 50 mM 2-deoxyglucose (2DG) to all samples (arrow). OCR traces are based on averaged technical triplicates ± SEM.

Supplemental Figure S7

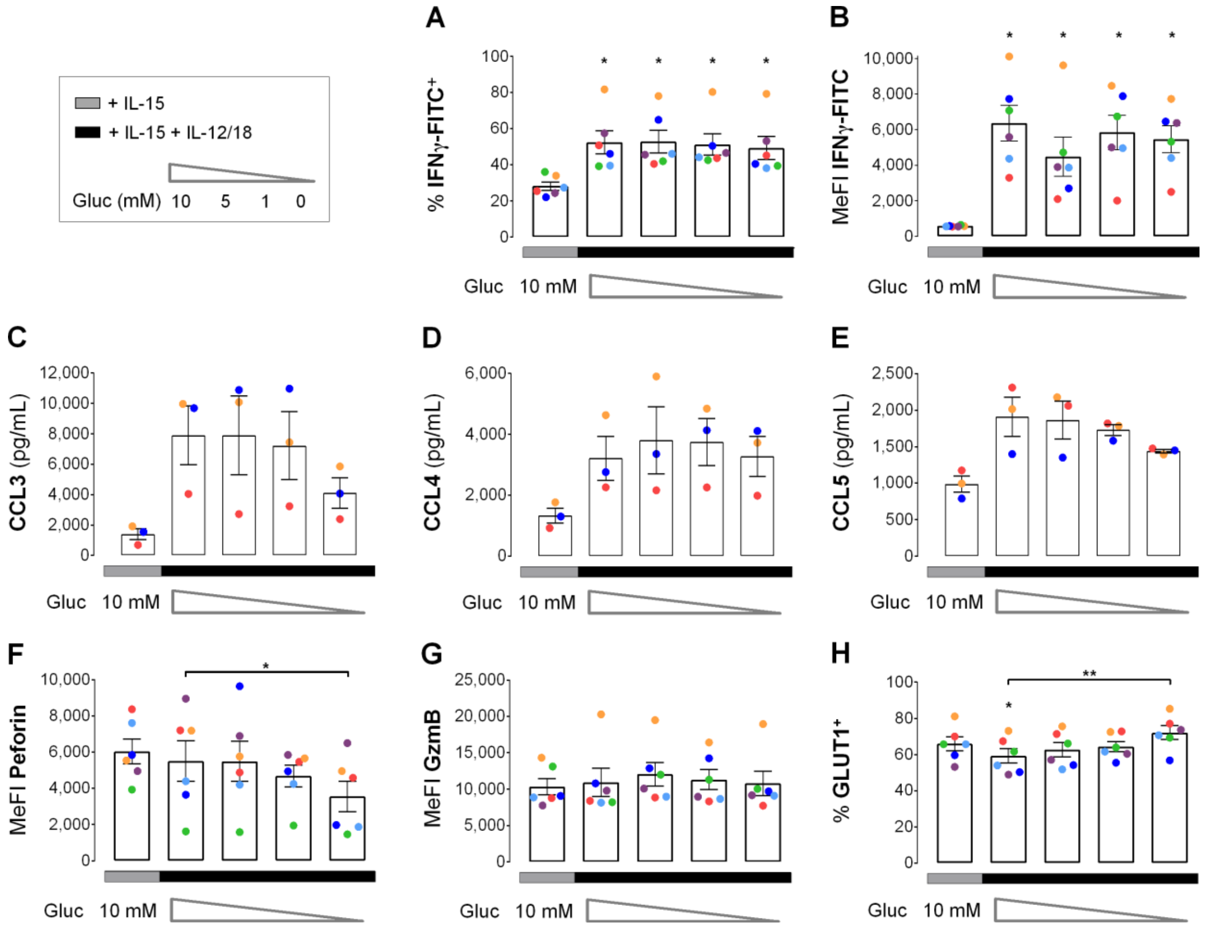

Early cytokine responses in human NK cells to IL-12/IL-18 after long-term IL-15 exposure do not require glucose. NK cells from 3 or 6 buffy coats were cultured for 120 h in the presence of IL-15 at a concentration of 100 ng/mL which was used throughout for IL-15 in this experiment. Cells were washed with glucose-deficient medium and were re-seeded either in fresh medium with IL-15 and glucose as a control (gray horizontal) or in medium containing IL-15 plus IL-12 and IL-18 (IL-12/18) at 10 and 50 ng/mL, respectively, and glucose concentrations declining from 10 mM to 0 mM (black horizontal). Four hours after re-seeding, cells were collected and analyzed for the proportion of (**A**) IFNγ$^+$ cells, (**B**) IFNγ MeFI, secreted (**C**) CCL3, (**D**) CCL4, and (**E**) CCL5, (**F**) perforin and (**G**) granzyme B (GzmB) MeFI, and (**H**) GLUT1$^+$ cells. Data is shown as mean values ± SEM (bars) and scatter plots in a color scheme to identify data from independent experiments. Within the IL-15 + IL-12/18 group, statistical significance of mean differences was determined with the Friedman test with Dunn's test for post-hoc pairwise comparisons. Each condition with IL-15 + IL-12/18 was compared individually to the IL-15 control with the Wilcoxon signed-rank test. * $p < 0.05$, ** $p < 0.01$.

Supplemental Figure S8

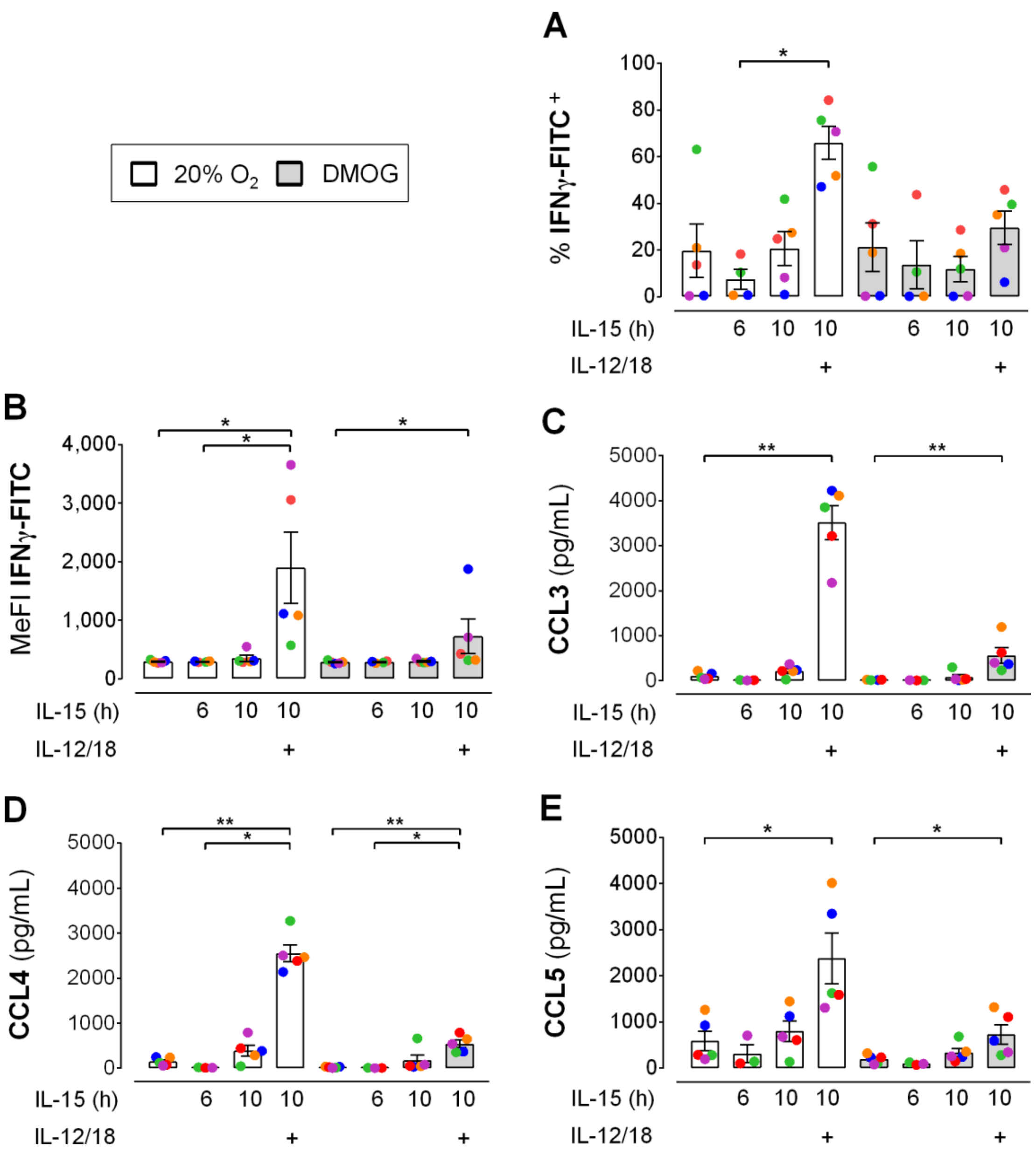

DMOG is detrimental to IFNγ production and CC chemokine release by IL-15 primed human NK cells in response to IL-12/IL-18. NK cells from 5 buffy coats were seeded and cultured in parallel under normoxia (20% O_2, open bars) and chemical hypoxia (DMOG, gray bars). IL-15 was added at 16 h and IL-12 and IL-18 (IL-12/18) at 22 h. Cells and supernatants were collected at either 22 h (6 h IL-15 only) or at 26 h (untreated controls and 10 h IL-15 with or without 4 h IL-12/18) and analyzed for (**A**,**B**) cellular IFNγ production and levels of secreted (**C**) CCL3, (**D**) CCL4, and (**E**) CCL5. Data is shown as mean values ± SEM (bars) and scatter plots in a color scheme to identify data from independent experiments. Within the normoxia and chemical hypoxia group each, statistical significance of mean differences was determined with the Friedman test with Dunn's test for post-hoc pairwise comparisons. * $p < 0.05$, ** $p < 0.01$. Corresponding treatments under normoxia and hypoxia were compared with the Wilcoxon signed-rank test but did not reach the level of statistical significance.

Supplemental Discussion

As the properties of NK cell immunometabolism are beginning to be unraveled, distinct "metabolic fingerprints" were recently proposed to underpin NK cell functional states [65]. Accordingly, NK cell polarization to a cytotoxic phenotype is distinguished by glycolytic and oxidative metabolism, to a memory phenotype by enhanced mitochondrial fitness, and to a regulatory phenotype by metabolic adaption to hypoxic and glucose-limiting conditions. Apparently, NK cells adopt more than one of these functional states in the course of an immune reaction. First, they sense inflammation and translocate to the site of its origin, then eliminate cells showing signs of stress due to, e.g., infection or transformation, and support transition to an adaptive immune response and resolution of inflammation. The timing for the display of a cytotoxic and memory NK phenotype and according metabolic features may be relatively obvious, but regulatory functions can be identified throughout the inflammatory reaction. As features of inflamed tissues, hypoxia and low glucose, that are supposedly associated with a regulatory phenotype, eventually need to resolve to allow for an efficient adaptive immune response and resolution of inflammation. NK cells may hence be expected to be best adapted to these conditions before they contact and attack stressed cells, i.e., the scenario that our in vitro IL-15 priming and secondary IL-12/IL-18 stimulation regiment attempts to mimic.
